# Isolation and Screening of Antagonistic Endophytes against *Phytophthora infestans* and Preliminary Exploration on Anti-oomycete Mechanism of *Bacillus velezensis* 6-5

**DOI:** 10.3390/plants12040909

**Published:** 2023-02-17

**Authors:** Jiaomei Zhang, Xiaoqing Huang, Yuqin Hou, Xiangning Xia, Zhiming Zhu, Airong Huang, Shun Feng, Peihua Li, Lei Shi, Pan Dong

**Affiliations:** 1 School of Life Sciences, Chongqing University, Chongqing 401331, China; 2 Chongqing Key Laboratory of Biology and Genetic Breeding for Tuber and Root Crops, Chongqing 400716, China; 3 College of Agronomy, Xichang University, Xichang 615013, China

**Keywords:** biological control, *Phytophthora infestans*, endophytes, *Bacillus velezensis*, potato late blight

## Abstract

*Phytophthora infestans*, the notorious pathogen of potato late blight, leads to a severe decline in potato yields and even harvest failure. We isolated 201 endophytic isolates from healthy root tissues of potatoes, among which 41 showed strong antagonistic activity against *P. infestans*. Further, the tolerance to stress and the potential application against potato late blight of these antagonistic isolates were tested. Most of them were extremely tolerant to stresses such as acid–alkali, temperature, UV, salt, and heavy metal stress. However, some antagonistic isolates with excellent stress tolerance might be pathogenic to potatoes. Combining the screening results, a total of 14 endophytes had excellent comprehensive performance in all the tests. In this paper, the endophyte 6-5 was selected among them for the preliminary exploration of the anti-oomycete mechanism. Analysis of the 16S rDNA sequence revealed that 6-5 had a high homology to the corresponding sequence of *Bacillus velezensis* (99.72%) from the NCBI database. Endophyte 6-5 significantly inhibited the mycelial growth of *P. infestans*, with an inhibition rate of over 90% in vitro assays, and deformed the hyphal phenotype of *P. infestans*. In addition, endophyte 6-5 could secrete protease and cellulase, and produce antagonistic substances with high thermal stability, which might be helpful to its antagonistic activity against *P. infestans*. Furthermore, it was demonstrated that 6-5 had the ability to improve the resistance of potato tubers to late blight. In short, our study described the process of isolating and screening endophytes with antagonistic activity against *P. infestans* from potato roots, and further explored the potential of biocontrol candidate strain 6-5 in potato late blight control.

## 1. Introduction

The potato (*Solanum tuberosum* L.) serves as one of the most important global crops, ranking fourth after wheat, rice, and maize [1]. A potato has extremely high nutritional value and unique flavor, and its tuber is rich in large quantities of starch, amino acids, and vitamins [2]. According to the Food and Agriculture Organization (FAO), potato production was estimated to exceed 359 million tons in 2020 [3]. However, the diseases that occur during crop growth are often a main constraint to its production, causing a significant decrease in production and food security threats [4]. Late blight, caused by *Phytophthora infestans*, is one of the most devastating diseases that significantly impact potato production worldwide [5]. Conservatively, the annual global economic costs associated with potato damage and disease control of late blight were estimated at more than USD 6 billion [6]. Since its earliest epidemic outbreak in Ireland in the nineteenth century, humans have never stopped searching for strategies to combat *P. infestans*, but most attempts have not been sufficiently effective [7]. Chemical control by spraying pesticides is the oldest method used against late blight and is still widely used currently [8]. Whilst pesticides are efficient at controlling this disease, they have certain drawbacks: an environmental and economic burden, food safety problems and human health threats, and the opportunity for drug-resistant strains to emerge [7,9,10]. Considering the threat to the environment, the EU has implemented directives on reducing the use of synthetic pesticides and increasing sustainable alternative disease control strategies [11]. As a result, there is an urgent need to develop alternative methods to reduce the use of pesticides.

Biological control, an alternative to pesticides, can be defined as the application of beneficial microorganisms to counteract plant pathogens, to reduce the symptoms of diseases [11,12]. Endophytes refer to microbes that colonize plant tissues or organs for part or all of their life cycles without causing adverse plant symptoms, including endophytic fungi, bacteria, and actinomyces [13,14]. Endophytes, by inducing plant resistance, competing for living space and nutrients with pathogens, or secreting secondary metabolites to inhibit the growth of pathogens, achieve the purpose of disease control [14,15,16,17]. In recent years, there has been a growing interest in the role of endophytes as biological control agents, because of their typical advantages such as variety, environmental friendliness, and non-pathogenicity [18]. For example, endophytic *Bacillus subtilis* L1-21 isolated from healthy citrus plants was reported to control huanglongbing (HLB), a devastating citrus disease, by inhibiting its pathogen, *Candidatus Liberibacter* asiaticus (*C*Las) [19,20]. *Bacillus velezensis* K1, an endophytic bacterium originally isolated from aerial roots of *Ficus benghalensi*, could inhibit 14 different fungal pathogens, including *Fusarium oxysporum*, *Mucor indicus*, *Sclerotia rolfsii*, etc. [21]. *Aspergillus fumigatus* LN-4, an endophytic fungus isolated from *Melia azedarach*, secreted alkaloids that displayed varying degrees of antifungal activities against *Fusarium oxysporum*, *Botrytis cinerea*, *Colletotrichum gloeosporioides* and so on [22]. Despite a large body of research that has isolated endophytes with biological control potential, relatively few endophytes were proven to be effective against late blight. This phenomenon may be explained by the fact that there are still a large number of endophytes with biocontrol potential of potato late blight yet to be isolated and identified [18]. Introducing endophytes as biological control agents might represent a sustainable and reliable attempt to replace pesticides in late blight management. Hence, the present study aimed to isolate, screen, and identify endophytes that have strong antagonism to *P. infestans* from healthy potato roots to be applied to the control of late blight in practical agricultural production in the near future.

## 2. Results

### 2.1. Isolation of Endophytes from Potato Roots and Screening of Endophytes with Anti-oomycete Activity against P. infestans

A total of 201 endophytic isolates were obtained from healthy potato root tissues, among which 41 could almost completely inhibit the growth of *P. infestans* T30-4 (Figure S1). These antagonistic isolates were selected as candidate isolates for subsequent studies.

### 2.2. Stress Tolerance Analysis of Candidate Isolates

Acid–alkali stress analysis showed that most of the candidate isolates grew well under the conditions of pH 5–9, suggesting they have certain acid–alkali tolerance. For temperature stress tolerance testing, almost all the candidate isolates could grow well in the temperature range of 17–57 °C. However, at 7 °C, their growth was almost completely inhibited. This implied these candidate isolates have better tolerance to high-temperature stress than low-temperature stress. In salt stress treatment, a concentration range of 0–30% (*w*/*v*) NaCl was added into the medium. It was found that all candidate isolates grew normally on all plates, indicating that they have a strong ability to resist salt stress by observing their growth on these salt-containing plates. Next, 5–80% (*w*/*v*) chromium ion (Cr^3+^) was designed to detect the resistance of candidate isolates to heavy metal stress. The results indicated that most of them grew well at 10% Cr^3+^, and more than two-thirds of the isolates could withstand 20% Cr^3+^ stress. Moreover, some isolates even tolerated 40% (*w*/*v*) Cr^3+^ stress, such as 13-2, 18-7, 12-15, H17-6, etc., which means that some candidate isolates had excellent tolerance to heavy metal stress. Finally, the UV stress test showed all the candidate isolates still maintained good growth states even under the longest UV irradiation time (40 min), which preliminarily indicated that all the candidate isolates could tolerate certain UV stress. In short, most of the candidate strains have good tolerance to salt stress, UV stress, heavy metal stress, acid–alkali stress, and high–low temperature stress, which makes them have potential as biocontrol microorganisms. According to these results, we screened a total of 32 isolates with good stress tolerance from 41 candidate isolates, as described in Table 1.

### 2.3. The Potential Application of Candidate Isolates against Potato Late Blight on Potato Tubers

**Figure 1:**
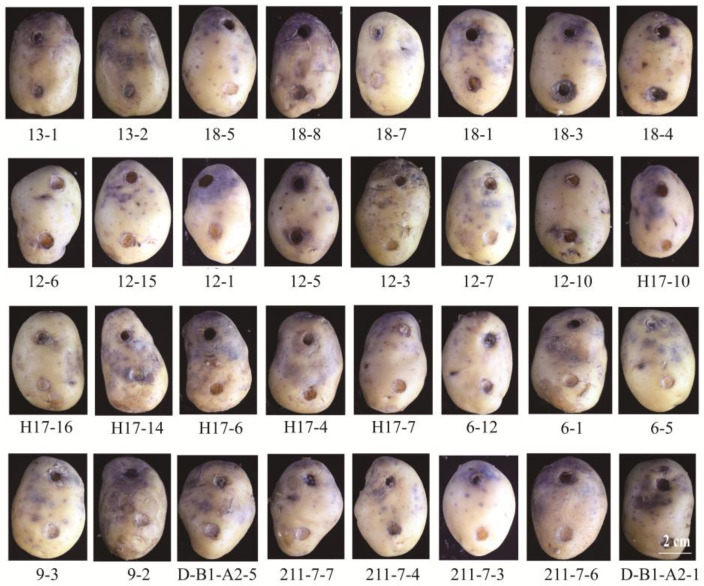
The growth of endophytic candidate isolates and *Phytophthora infestans* T30-4 on the potato tubers. *P. infestans* T30-4 was inoculated in the upper hole of the potato, and the endophytic candidate isolate was inoculated in the lower hole.

If these candidate isolates are to be applied to the control of potato late blight in practical production, it is critical to observe their effects on potato tubers during late blight development. Therefore, the effect of 32 isolates with good stress tolerance on potato tubers was further tested. The results suggested that over half of the potato tubers had lesions in the small holes where the candidate isolates had been inoculated, which might be caused by the pathogenicity of candidate isolates themselves to potatoes, or they could not inhibit the development of late blight well. Thus, these endophytes, such as 13-1, 12-5, 18-3, 18-4, D-B1-A2-1, etc., were not considered suitable for late blight control. Nevertheless, the rest were not pathogenic to potato tubers and could inhibit the development of late blight to a certain extent, such as 6-5, 6-1, etc., suggesting that further research on these isolates is quite necessary and valuable (Figure 1).

Comprehensively considering the results of the above isolation and screening tests, a total of 14 endophytic isolates with strong antagonistic activity against *P. infestans* T30-4, excellent stress tolerance, and no pathogenicity to potatoes were obtained. They are 18-5, 18-7, 12-3, 12-7, H17-10, H17-16, H17-6, H17-7, 6-1, 6-5, 9-3, 211-7-7, 211-7-3, and 211-7-6. For practical application in late blight management, further research on these isolates is quite necessary. In this study, we only selected an endophyte 6-5 among them for subsequent studies to preliminary explore its anti-*P. infestans* mechanisms.

### 2.4. Identification of Endophytes 6-5

The morphological characteristics of endophyte 6-5 could be described as milky white, nearly spherical, viscous, with a neat edge and a smooth surface of the colony (Figure S2A). The results of physiological and biochemical characterization revealed that 6-5 is an aerobic Gram-positive bacterium with the ability to produce gelatinase, catalase, and amylase, and reduce nitrate (Figure S2B). Additionally, the 16S rDNA sequence of 6-5 was obtained for sequence homology and phylogenetic analysis, and the results showed that 6-5 was very closely related to *Bacillus velezensis* GD-1, with a homology of 99.72% (Figure S2C). Based on colony morphology, physiological and biochemical characteristics, and the analysis of the 16S rDNA sequence, endophyte 6-5 was speculated as *B. velezensis*.

### 2.5. Effect of 6-5 on Mycelial Growth, Hyphal Phenotype, and Spore Germination of P. infestans

**Figure 2:**
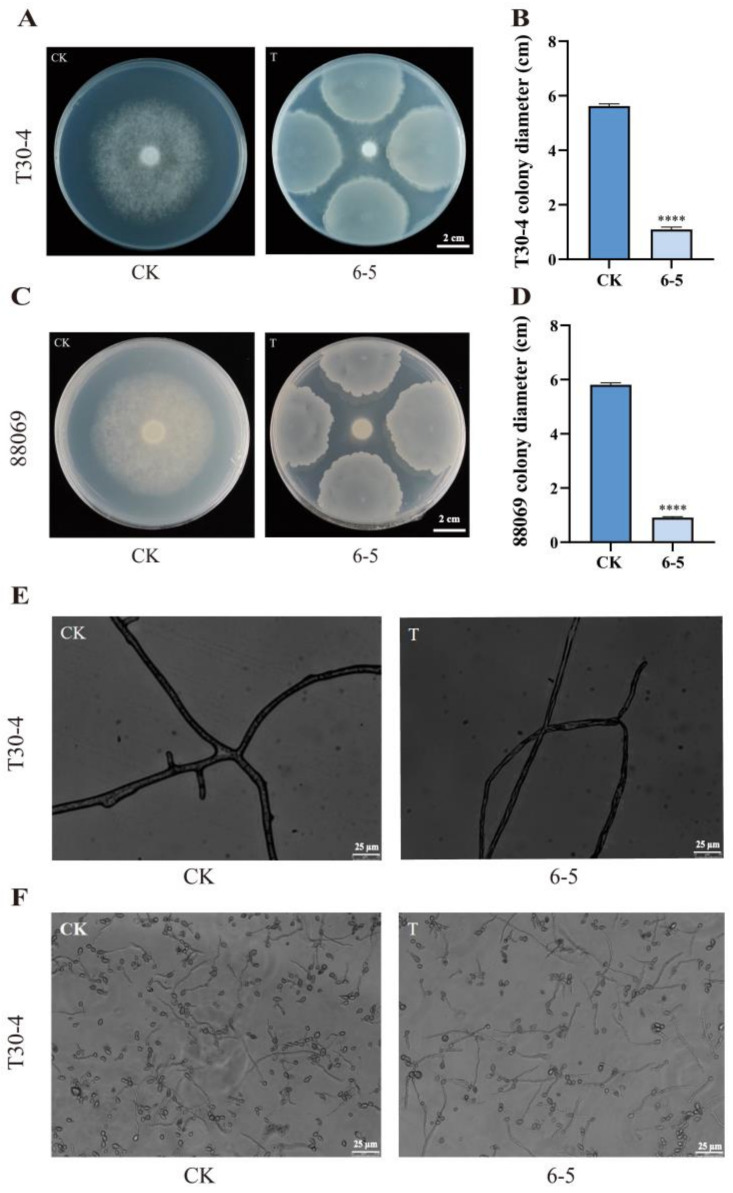
Effect of 6-5 on mycelial growth, hyphal phenotype, and spore germination of *Phytophthora infestans*. (**A**) Colony morphology of strain T30-4 under 6-5 confrontation; (**B**) colony diameter of strain T30-4 under 6-5 confrontation; (**C**) colony morphology of strain 88069 under 6-5 confrontation; (**D**) colony diameter of strain 88069 under 6-5 confrontation; (**E**) hyphal phenotype of T30-4 treated with 6-5 under the microscope; (**F**) the spore germination of T30-4 observed under a microscope after CK or 6-5 treatment. **** *p* < 0.0001.

To evaluate the inhibitory effects of 6-5 on mycelial growth of *P. infestans* by the dual culture assay. We found that mycelial growth of the T30-4 strain (A1 mating type) and 88069 strain (sterile mating type) were almost completely inhibited under 6-5 confrontation. After 7 d of culture, the colony diameters of the T30-4 strain and 88069 strain were 1.1 cm and 0.91 cm, respectively, which were significantly lower than the control (Figure 2A–D). The inhibition rates of T30-4 and 88069 were calculated as 91.89% and 95.89%, respectively.

Microscopic observation showed that the hyphae of *P. infestans* by the treatment of 6-5 were slender with more vacancies, and the hyphae widths were nonuniform, while the hyphae in the control group grew normally (Figure 2E). The effect of 6-5 on the spore germination of T30-4 was observed under the microscope. It was found that the spore germination of T30-4 with 6-5 treatment was decreased compared with the control, but the decrease was not significant (Figure 2F). These results indicated that 6-5 could markedly inhibit the mycelial growth of *P. infestans* (T30-4 and 88069) and deform the hyphal phenotype of T30-4, but its effect on T30-4 spore germination was not obvious.

### 2.6. Detection of Extracellular Enzymes of 6-5 and Localization of Antagonistic Substances of 6-5 against P. infestans

**Figure 3:**
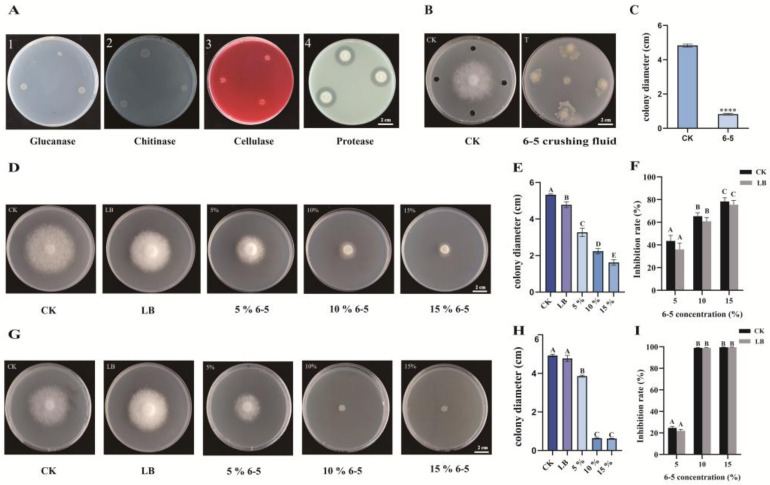
Detection of extracellular enzymes of 6-5 and localization of antagonistic substances of 6-5 against *Phytophthora infestans*. (**A**) The ability of 6-5 to secrete extracellular enzymes; (**B**) colony morphology of strain T30-4 under the treatment of 6-5 bacterial crushing fluid; (**C**) the colony diameter of strain T30-4 under the treatment of bacterial crushing fluid; (**D**) growth of strain T30-4 under different concentrations of 6-5 cell-free culture filtrate (filter sterilization); both CK and LB were set as controls, CK represents ordinary rye agar medium, and LB represents rye agar medium added with 10% LB. (**E**) the colony diameter of strain T30-4 under different concentrations of 6-5 cell-free culture filtrate (filter sterilization). (**F**) the inhibition rate of strain T30-4 by different concentrations of cell-free culture filtrate (filter sterilization). CK: the inhibition rate was calculated by comparing with the CK group; LB: the inhibition rate was calculated by comparing with the LB group. (**G**) growth of strain T30-4 under different concentrations of 6-5 cell-free culture filtrate (autoclaving); both CK and LB were set as controls, CK represents ordinary rye agar medium, and LB represents rye agar medium added with 10% LB. (**H**) the colony diameter of strain T30-4 under different concentrations of 6-5 cell-free culture filtrate (autoclaving). (**I**) the inhibition rate of strain T30-4 by different concentrations of cell-free culture filtrate (autoclaving). CK: the inhibition rate was calculated by comparing with the CK group; LB: the inhibition rate was calculated by comparing with the LB group. **** *p* < 0.0001, capital letters indicate a significant difference (*p* < 0.01).

In the extracellular enzymes production assay, there was transparent circle appeared around the colony of 6-5 on the CMC-Na medium and skim milk medium, while it was not observed on the chitin medium and poria cocos medium (Figure 3A). The phenomena implied that 6-5 has the ability to secrete extracellular enzymes, including cellulase and protease, but excluding chitinase and glucanase.

To locate antagonistic substances of endophyte 6-5 against *P. infestans*, bacterial crushing fluid and cell-free culture filtrate of 6-5 were obtained and used to treat *P. infestans*, separately. The mycelial growth of *P. infestans* was markedly inhibited by bacterial crushing fluid, and the colony diameter of *P. infestans* was only 0.82 cm, while it was 4.83 cm without treatment (Figure 3B,C). On the rye agar medium supplemented with 5%, 10%, and 15% (*v*/*v*) cell-free culture filtrate (filter sterilization), the higher the concentration of cell-free culture filtrate was, the slower the mycelial growth of *P. infestans* was, which showed a dose-dependent effect. With the 15% (*v*/*v*) cell-free culture filtrate treatment, the growth of *P. infestans* was strongly inhibited, and the inhibition rate reached over 75% after 7 d of cultivation (Figure 3D–F). In addition, the cell-free culture filtrate, obtained by autoclaving at 121 °C, also showed significant inhibitory activity on *P. infestans*, with an inhibition rate of more than 99% at 15% concentration after growing 5 d (Figure 3G–I). These results suggested that antagonistic substances of 6-5 against *P. infestans* exist in both intracellular and extracellular, and extracellular antagonistic substances have high thermal stability.

### 2.7. Effect of 6-5 on Inducing the Resistance of Potato Tubers to Late Blight

**Figure 4:**
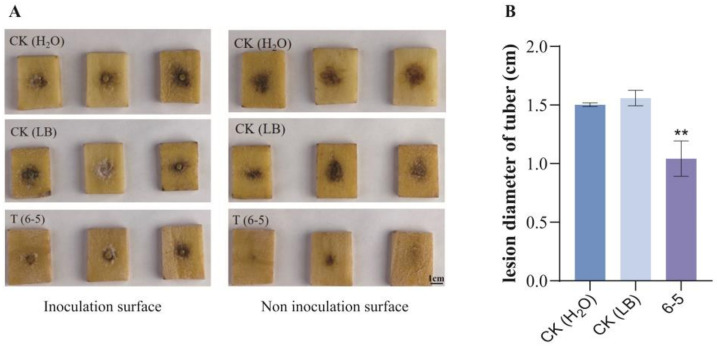
Effect of 6-5 on the resistance of potato tubers to late blight. (**A**) The disease symptom of potato tuber slices pretreated with 6-5 bacterial suspension; (**B**) the lesion diameter of potato tuber slices. ** *p* < 0.01.

To evaluate whether 6-5 can enhance the resistance of potatoes to late blight, *P. infestans* T30-4 was inoculated on potato tuber slices pretreated with 6-5 bacterial suspensions. The lesion diameter of potato tuber slices in the treatment group was 1.04 cm, compared with 1.50 cm in the CK (H_2_O) group and 1.56 cm in the CK (LB) group (Figure 4A,B). The result demonstrated that 6-5 could reduce the infection of *P. infestans* and alleviate the symptoms of potato late blight by improving the resistance of potato tubers.

## 3. Discussion

Endophytes, natural resources for plant disease control, can enhance plant resistance and inhibit the growth of phytopathogens, which has been proved by current research reports [23,24,25]. The isolation and screening of endophytes with antagonistic properties against pathogens are indispensable first steps for potential biocontrol endophytes in the search [26]. Endophytes can be isolated from the vegetative and reproductive organs of plants, among which the roots are the most important sources of endophytes, containing the largest number and the most abundant species [27,28]. In addition, endophytes from healthy plants in habitats with disease problems are more likely than introduced species to become competitive biological control microorganisms due to environmental adaptation [11]. Grace Ngatia et al. isolated 357 endophytes from four *solanaceous* plants in Kenya; only a limited proportion of approximately 13% of the isolates showed potential activity against *P. infestans* with a maximum inhibition rate of nearly 85%. Additionally, it was worth noting that 63% of the endophytes were obtained from Kilifi, a non-potato growing area, implying that regional isolation may limit pathogen–antagonist interaction [29]. Based on these facts, we harvested healthy potato root tissues from the fields of Wuxi County, a main potato-producing area, where potato late blight occurs naturally in successive years in Chongqing, China. A total of 201 endophytes were isolated from the healthy potato root tissues, among which 41, accounting for 20.4% of the total isolates, almost completely inhibited the growth of *P. infestans*. Our results suggested that it was extremely critical to select suitable host tissues and host planting sites for the isolation of large and abundant endophytes that are antagonistic to pathogens.

Endophytes with better survival and adaptation against abiotic stress are always associated with better biological control performance [30]. Moreover, endophytes with excellent stress tolerance can enhance plant resistance to abiotic stresses such as drought, heavy metals, salt stress, etc., and greatly promote the increase in crop yield in agricultural production [31,32,33]. Thus, in this study, 41 antagonistic isolates were further tested for stress tolerance analysis to screen isolates with strong resistance to stress. The results showed that 32 of them have excellent tolerance to salt, UV, heavy metal, acid–alkali, and high–low temperature stress, which might be one of the reasons for their antagonistic activity against *P. infestans*. Similarly, for pathogens, the ability to colonize plants is greatly limited by the environment. For instance, the successful epidemic of potato late blight often requires favorable environmental conditions [34,35]. The mycelial growth, spore production, and spore release of *P. infestans* were inhibited, causing a significantly reduced success rate of infection ultimately under adverse stress such as salt, high temperature, low temperature, UV, and so on [36]. Based on the strong resistance to stress of these candidate isolates and the environmental sensitivity of *P. infestans*, it is considered possible to combine these antagonistic isolates with stress conditions to control late blight in the future. Additionally, UV, salt, temperature, and other methods combined with biocontrol microorganisms have been proven to have a synergistic effect on the inhibition of pathogens, which further provided a basis for joint application [37,38]. For biocontrol microorganisms to be applied in practical production, it is necessary to ensure that they are non-pathogenic to crops. Therefore, we carried out a potato tuber test of 32 antagonistic isolates for the last step of screening. It was found that over half of them were pathogenic to potatoes or difficult to inhibit late blight development, and they would be excluded from the candidate isolates for biological control of potato late blight. Our study confirmed that this screening step is critical and essential, but is rarely considered in similar research. Comprehensively considering the results of screening tests, we selected 14 endophytes with strong antagonistic activity against *P. infestans*, excellent stress tolerance, and no pathogenicity to potatoes for subsequent studies. In this paper, we described the preliminary investigation of anti-*P. infestans* mechanism of endophytes 6-5.

Endophytes 6-5 were speculated as *B. velezensis*, a Gram-positive bacterium with strong stress tolerance. *B. velezensis* has been reported to exhibit antagonistic activity against a wide range of phytopathogens, considered one of the most common biocontrol bacteria [39,40,41,42]. For example, *B. velezensis* OEE1, isolated from root tissues of olive trees, can significantly suppress *Verticillium* wilt of olive [43]. *B. velezensis* KOF112 had antagonistic activities against gray mold caused by *Botrytis cinerea*, anthracnose by *Colletotrichum gloeosporioides*, and downy mildew by *Plasmopara viticola* [44]. Currently, a few *B. velezensis* are commercialized as efficient biocontrol agents, include *B. velezensis* FZB42, *B. velezensis* 9912D, *B. velezensis* SQR9 [21,42]. In Vitro, 6-5 could significantly inhibit the mycelial growth of different physiological races T30-4 and 88069, with inhibition rates of 91.89% and 95.89%, respectively. Previously, *B. velezensis* FZB42 has been demonstrated to significantly inhibit the growth of a variety of *Phytophthora* species, including *Phytophthora sojae* and *P. infestans*, which is similar to our result [45].

Bacterial extracellular hydrolytic enzymes such as protease, cellulase, chitinase, and glucanase were involved in the biocontrol of pathogens through degrading cell walls [46,47,48]. For instance, *Trichoderma atroviride* produced a large number of proteases and cellulases when in a dual culture with *Phytophthora cinnamomic* (a devastating widespread invasive oomycete), which is one of the several mechanisms known to be involved in *Trichoderma* biological control ability [49,50,51]. In addition, cellulase also plays a critical role in the dissolution of plant cell walls or plant tissues that may help endophytes enter or colonize the host tissues [4,30]. In the extracellular enzymes assay, we detected that 6-5 secreted protease and cellulase on the plates, suggesting the production of these extracellular enzymes may be one of the biocontrol mechanisms of 6-5.

The production of antagonistic substances, such as hydrolase, alkaloids, antibiotics, and volatile compounds, is an important antimicrobial mechanism of biocontrol microorganisms [14,52,53]. *Bacillus* species could secret various types of biologically active substances that significantly control and inhibit the growth of pathogens [30,54,55]. *B. velezensis* FZB42 has been reported to produce more than 13 antimicrobial compounds, such as bacillaene, difficidin, macrolactin, etc. [45]. In our study, it was found that antagonistic substances of 6-5 against *P. infestans* exist in both intracellular and extracellular, and extracellular antagonistic substances have high thermal stability. Antagonistic substances with high thermal stability can enhance the effectiveness of practical applications. Therefore, these antagonistic substances should be further extracted and analyzed in subsequent studies to be applied to practical production.

Some studies have shown that endophytes can enhance host resistance to diseases. Kazuhiro Hamaoka et al. found endophyte *B. velezensis* KOF112 induced grapevine defense response through both salicylic acid- and jasmonic acid-dependent defense pathways [44]. Endophytes *B. velezensis* BBC023 and BBC047 can produce surfactin, which induces systemic resistance of tomato plants against *B. cinerea* [56]. In potato tuber assays, after potato tubers were pretreated with 6-5, the infection of *P. infestans* on potato tubers was significantly reduced, and the symptoms of the disease were alleviated, suggesting that 6-5 could successfully induce the resistance of potato tubers to potato late blight. However, the effect of the practical application still needs to be further tested in the field.

## 4. Materials and Methods

### 4.1. Materials

The potatoes (variety: Favorita) were collected from the fields of Dabao Village, Jianshan Town, Wuxi County, the main potato-producing area in Chongqing City, China (109°63′ E longitude and 31°40′ N latitude). Endophytes were isolated from healthy root tissues of potatoes. Two different mating types of *P. infestans* strains were tested: *P. infestans* strain T30-4 (A1 mating type) was kindly provided by Professor Suomeng Dong of Nanjing Agriculture University, China; *P. infestans* strain 88069 (sterile mating type) was kindly provided by Professor Jiasui Zhan of Fujian Agriculture and Forestry University, China. The strains were incubated on rye agar medium at 20 °C in the dark. LB agar plates and PDA agar plates were used for the culture and screening of bacteria and fungi, respectively. Chemicals used for extracellular enzyme assay and physiological and biochemical were purchased from Sangon Biotech (Shanghai, China).

### 4.2. Isolation of Endophytes from Roots of Potato

The potato root tissues were washed thoroughly with sterile water to remove soil and impurities from the surface. Then, the surface was sterilized by immersion in 70% alcohol for 5 min. Subsequently, sterilize using 10% sodium hypochlorite (NaClO) solution for 5 min, and then rinse with sterile double distilled water thrice. The final rinse solution was collected and coated on LB and PDA agar plates, and each was coated with 0.5 mL solution. Then, LB and PDA agar plates were, respectively, cultured inverted at 37 °C and 28 °C for 7 d to confirm the surface sterilization efficiency of root tissues. Under a sterile environment, surface sterilized root segments with 0.9% sterile normal saline were homogenized by making a paste using a mortar and pestle, which were incubated at 28 °C and 180 r/min for 40 min. After appropriate dilution, the tissue suspensions (10^−1^–10^−4^) were further coated on LB or PDA agar plates and incubated at 37 °C or 28 °C, respectively. The plates were thoroughly checked daily to monitor the growth of endophytes. After the appearance of the colonies, they were selected and purified by the streak plate method, based on their morphological and color differences. Endophytic isolates were continually transferred to new plates and further re-streaked until pure colonies were achieved. Purified single colonies were then stored at 4 °C for further analysis.

### 4.3. Screening of Endophytes with Anti-oomycete Activity against P. infestans

The dual culture assay was performed to test the antagonistic activity of endophytic isolates against *P. infestans*. A 7-mm-diameter *P. infestans* T30-4 mycelial disk (10 d old) was placed in the center of the rye agar medium. Then, the endophytic isolates were inoculated at equal distances (2.0 cm) around the *P. infestans* disk, while *P. infestans* grown in the plates without endophytes served as the negative control. After growing at 20 °C for 5 d, the growth state of the *P. infestans* colony was observed.

### 4.4. Stress Tolerance Analysis of Candidate Isolates

The endophytic isolates with antagonistic activity against *P. infestans* were selected as candidate isolates, and their stress tolerance was tested. Five types of stresses conditions were set, and detailed treatments were as follows:

(1) Salt stress: candidate isolates were successively coated on LB and PDA agar plates containing 0, 0.01, 0.05, 0.1, 0.3 (*w*/*v*) NaCl. (2) Heavy metal stress: candidate isolates were inoculated on LB and PDA agar plates supplemented with chromium ion (0, 5%, 10%, 20%, 40%, 80% *w*/*v*). (3) Acid–alkali stress: the pH values of LB and PDA agar plates were adjusted to 5, 7, 9, and 13, respectively, with 0.1 M HCl/NaOH. Next, candidate isolates were cultured on those plates with different pH values. (4) Temperature stress: candidate isolates were cultivated on LB and PDA agar plates at 7 °C, 17 °C, 27 °C, 37 °C, 47 °C and 57 °C for 12 h. (5) Ultraviolet stress: LB and PDA agar plates inoculated with candidate isolates were exposed to UV light for 5 min, 10 min, 15 min, 20 min, and 40 min. All plates except those treated with temperature stress were placed at 37 °C (LB) and 28 °C (PDA) for 12 h.

### 4.5. Potato Tubers Test of Candidate Isolates

Further, antagonistic endophytic isolates with good stress tolerance for potato tuber test were selected. Healthy, solid, and intact potato tubers were selected, and their surfaces were sterilized as described by Feng et al. [57]. After their surfaces were sterilized, they were washed with sterile water three times, and then put on sterile filter paper to dry naturally. Considering the interaction between *P. infestans* and antagonistic endophytic isolates on the potato tubers, two 7-mm-diameter holes were carved into the potato tubers, and the distance between them was 4 cm. Then, a 6-mm-diameter *P. infestans* T30-4 mycelial disk (10 d old) was inoculated to one hole, and candidate isolate (OD_600_ = 0.2) was added to the other [57,58]. Place inoculated potato tubers on a stainless tray (60 cm × 40 cm × 5 cm) with sterile water-soaked filter paper to ensure humidity and cover with plastic wrap. After five days of incubation at 20 °C, the effect of the candidate isolates on potato tubers was observed during the development of potato late blight.

### 4.6. Identification of Endophytic Isolate 6-5

Morphological identification was conducted by observing the single-colony related characteristics (color, shape, edge state, surface texture, viscosity, etc.). Physiological and biochemical identification was performed in accordance with Berger Bacterial Identification Manual and Common Bacterial System Identification Manual [59,60]. Molecular identification by 16s rDNA analysis: Total genomic DNA of endophyte 6-5 was extracted using Bacterial Genomic DNA Extraction Kit (Omega BioTek, Inc., Norcross, GA, USA), and then the 16S rDNA was amplified using universal primer 27F/1492R. The 16s rDNA amplified fragment was sent to Tsingke Biotechnology Co., Ltd., China, for sequencing. Nucleotide sequence homology inquiries were performed through the NCBI (https://www.ncbi.nlm.nih.gov/, accessed on 2 October 2021.) BLAST program. Furthermore, the Clustal X was used to make multiple sequence alignments, and the Neighbor-joining method was employed to construct the phylogenetic tree by MEGA-X software. The 16S rRNA gene sequences of 6-5 were submitted to NCBI GenBank and assigned the GenBank accession number OQ421469.

### 4.7. Effect of 6-5 on Mycelial Growth, Hyphal Phenotype, and Spore Germination of P. infestans

Antagonism of endophyte 6-5 against mycelial growth of different physiological races of *P. infestans*, T30-4 and 88069, was tested by the dual culture assay. On the center of the rye agar medium (90 mm), a 7-mm-diameter *P. infestans* mycelial disk (10 d old T30-4 or 88069) was placed, and then the 6-5 was inoculated at equal distances around the disk (25 mm). The plates were only inoculated with 88069 or T30-4 as a control. After 7 d of dark growth at 20 °C, the final colony diameter of *P. infestans* was measured with a ruler (cross method), and the inhibition rate was calculated. Further, the hyphae of T30-4 cultured with or without 6-5 confrontation were taken under the microscope to observe the phenotype. The spore suspension of *P. infestans* was obtained by rinsing plates covered with *P. infestans* T30-4 mycelia with sterilized water. Then, 0.5 μL 6-5 suspension was added to 200 μL T30-4 spore suspension, and the same volume of LB broth was added as the control. The spores were cultured at 20 °C for 12 h, and the spore germination was observed under the microscope. The microscope used for observation of spore germination and hyphal phenotype was Optiplex 3050 (Dell, Inc., Round Rock, TX, USA) inverted fluorescence phase contrast microscope.

Inhibition rate (%) = (diameter of the colony in the control plate—diameter of the colony in the treatment plate)/(diameter of the colony in the control plate—initial colony diameter) × 100%.

### 4.8. Detection of Extracellular Enzymes of 6-5

The following plates were prepared for the detection of extracellular enzymes: (1) Chitin medium was prepared with 5 g/L chitin, 0.5 g/L yeast extract, 0.5 g/L K_2_HPO_4_, 0.2 g/L MgSO_4_, 0.1 g/L NaCl and 15 g/L agar powder for chitinase detection; (2) Carboxymethyl cellulose sodium salt (CMC-Na) medium was prepared with 20 g/L CMC-Na, 1.5 g/L K_2_HPO_4_, 2.5 g/L Na_2_HPO_4_, 2.5 g/L tryptone, 0.2 g/L Congo red and 15 g/L agar powder to identify cellulase; (3) Poria cocos medium was prepared by adding 5 g/L yeast extract, 4 g/L poria cocos powder, 1.5 g/L K_2_HPO_4_, 2.5 g/L Na_2_HPO_4_, 0.1 g/L aniline blue and 15 g/L agar powder for detecting glucanase; (4) Protease production was monitored using skim milk medium (skim milk 250 mL, agar powder 7.5 g, distilled water 250 mL) [61]. Endophytic bacterium 6-5 was inoculated on these plates and cultured for 1 d at 37 °C to observe whether there were transparent circles around the colonies.

### 4.9. Localization of Antagonistic Substances of 6-5 against P. infestans

Endophyte 6-5 was inoculated in LB broth at 37 °C, while it was shaken at 180 rpm for 1 d to obtain 6-5 bacterial suspensions. Next, 6-5 bacterial suspensions were transferred into a 50 mL centrifuge tube and centrifuged at 10,000 RCF for 5 min, then the supernatant and the bacteria were collected, respectively. Next, the bacteria were resuspended with 10 mL PBS solution, broken by a sonicator for 30 min after the ice bath, centrifuged at 10,000 RCF for 2 min, and the supernatant was collected to obtain bacterial crushing fluid. The supernatant was sterilized in two different ways, one was autoclaving at 121 °C for 20 min, and the other was filter sterilization by a 0.22 µm microporous membrane to obtain the cell-free culture filtrate. The dual culture assay was carried out to observe the anti-*P. infestans* ability of 6-5 bacterial crushing fluid. The bacterial crushing fluid was added into four holes around a *P. infestans* T30-4 mycelial disk at equal distances, 20 μL per hole. Additionally, control was rye agar medium only inoculated with *P. infestans*. The plates were put at 20 °C for 5 d, then the colony diameter of *P. infestans* was measured by the cross method, and the inhibition rate was calculated. The effect of extracellular antagonistic substances on *P. infestans* was tested by inoculating a *P. infestans* T30-4 mycelial disk on the center of the rye agar medium, which was supplemented with cell-free culture filtrate (5%, 10%, 15% (*v*/*v*)). Rye agar medium added 10% LB and ordinary rye agar medium were used as negative controls. After growing at 20 °C for 5–7 d, the diameter of the colony was measured by the cross method, and the inhibition rate was calculated. The calculation of inhibition rate is the same as 4.7.

### 4.10. Effect of 6-5 on Inducing the Resistance of Potato Tubers to Late Blight

This test was performed as described by Elkahoui et al. [62], with some modifications. Potato tuber slices (4 cm × 3 cm × 8 mm in size) were soaked in 6-5 bacterial suspensions (10^7^ CFU/mL) for 20 min, and then washed with distilled water to remove the bacterial suspensions. Potato tuber slices soaked in sterile water and LB broth were used as controls. After the tuber slices were air-dried naturally, the 6-mm-diameter T30-4 mycelial disk was inoculated on potato tuber slices pretreated with 6-5 bacterial suspensions and cultured for 3–5 d under the conditions of avoiding light and moisturizing. Then, the lesion diameter of potato tuber slices was measured.

### 4.11. Statistical Analysis

Three biological repeats were performed for each experiment, and statistical analysis was conducted using *t*-test or Duncan’s analysis (GraphPad Prism v. 9.0.0 and IBM SPSS Statistics 22.0).

## 5. Conclusions

In this study, we isolated 201 endophytes from healthy root tissues of potatoes, among which 14 endophytes with strongly antagonistic activity against *P. infestans*, excellent stress tolerance, and no pathogenicity to potatoes. In addition, an antagonistic isolate 6-5, one of these 14 endophytes, was further tested for its anti-*P. infestans* ability and mechanism. Endophyte 6-5, speculated as *B. velezensis*, can strongly inhibit mycelial growth and alter the hyphal phenotype of *P. infestans*. Its anti-*P. infestans* mechanisms may include secretion of protease, cellulase, extracellular antagonistic substances with high thermal stability, and induction of potato tubers resistance to late blight. In conclusion, 6-5 might be an effective biocontrol bacterium for potato late blight control and anti-*P. infestans* mechanisms of other antagonistic isolates are waiting for subsequent studies.

## Figures and Tables

**Table 1:** Candidate isolates with good stress tolerance.

Candidate Isolates	pH 5	pH 9	57 °C	17 °C	UV 40 min	10% Cr^3+^	20% Cr^3+^	40% Cr^3+^	30% NaCl
13-1	++	++	++	+	++	++	++	−	++
13-2	++	++	++	++	++	++	++	++	++
18-5	++	++	++	++	++	++	++	−	++
18-8	++	++	++	++	++	++	−	−	++
18-7	++	++	++	++	++	++	++	++	++
18-1	++	++	++	++	++	++	++	−	++
18-3	++	++	++	++	++	++	++	−	++
18-4	++	++	++	++	++	++	++	−	++
12-6	++	++	++	++	++	++	++	−	++
12-15	++	++	++	++	++	++	++	++	++
12-1	++	++	++	++	++	++	++	−	++
12-5	++	++	++	++	++	++	++	++	++
12-3	++	++	++	++	++	++	−	−	++
12-7	++	++	++	++	++	++	++	−	++
12-10	++	−	++	++	++	++	++	−	++
H17-10	++	+	++	++	++	++	++	−	++
H17-16	++	++	++	++	++	++	++	−	++
H17-14	++	++	++	++	++	++	++	++	++
H17-6	++	++	++	++	++	++	++	++	++
H17-4	−	++	++	++	++	++	++	++	++
H17-7	−	++	++	++	++	++	++	++	++
6-12	++	++	++	++	++	++	++	−	++
6-1	++	++	++	++	++	++	++	−	++
6-5	++	++	+	+	++	++	++	−	++
9-3	++	++	++	++	++	++	++	−	++
9-2	++	++	++	++	++	++	−	−	++
D-B1-A2-5	++	++	++	++	++	++	++	−	++
211-7-7	++	++	++	++	++	++	++	−	++
211-7-4	−	++	++	++	++	++	++	−	++
211-7-3	++	++	++	++	++	++	++	−	++
211-7-6	−	++	++	++	++	++	−	−	++
D-B1-A2-1	++	++	++	++	++	++	++	−	++

Note: + means normal growth, and ++ means better growth; − means poor growth.

## Data Availability

The data presented in this study are available on request from the corresponding author.

## Supplementary Materials

The following supporting information can be downloaded at https://www.mdpi.com/article/10.3390/plants12040909/s1. Figure S1: Endophytic isolates with antagonistic activity against *Phytophthora infestans* T30-4 in vitro; Figure S2: Identification of endophyte 6-5. (A) Colony characteristics of 6-5; (B) Physiological and biochemical characteristics of 6-5, + indicates a positive reaction, − indicates a negative reaction; (C) The phylogenetic tree of 6-5.

## References

[1] Wu Z.H., Ma Q., Sun Z.N., Cui H.C., Liu H.R. (2021). Biocontrol mechanism of *Myxococcus fulvus* B25-I-3 against *Phytophthora infestans* and its control efficiency on potato late blight. Folia Microbiol..

[2] Kondhare K.R., Natarajan B., Banerjee A.K. (2020). Molecular signals that govern tuber development in potato. Int. J. Dev. Biol..

[3] Paluchowska P., Sliwka J., Yin Z. (2022). Late blight resistance genes in potato breeding. Planta.

[4] Wang Y., Pruitt R.N., Nurnberger T., Wang Y. (2022). Evasion of plant immunity by microbial pathogens. Nat. Rev. Microbiol..

[5] Sorokan A., Benkovskaya G., Burkhanova G., Blagova D., Maksimov I. (2020). Endophytic strain *Bacillus subtilis* 26DCryChS producing cry1Ia toxin from *Bacillus thuringiensis* promotes multifaceted potato defense against *Phytophthora infestans* (Mont.) de Bary and Pest *Leptinotarsa decemlineata* Say. Plants.

[6] Fry W.E., Birch P.R., Judelson H.S., Grunwald N.J., Danies G., Everts K.L., Gevens A.J., Gugino B.K., Johnson D.A., Johnson S.B. (2015). Five reasons to consider *Phytophthora infestans* a reemerging pathogen. Phytopathology.

[7] Ivanov A.A., Ukladov E.O., Golubeva T.S. (2021). *Phytophthora infestans*: An overview of methods and attempts to combat late blight. J. Fungi.

[8] Cohen Y., Rubin A.E., Galperin M. (2021). Effective control of two genotypes of *Phytophthora infestans* in the field by three oxathiapiprolin fungicidal mixtures. PLoS ONE.

[9] Axel C., Zannini E., Coffey A., Guo J., Waters D.M., Arendt E.K. (2012). Ecofriendly control of potato late blight causative agent and the potential role of lactic acid bacteria: A review. Appl. Microbiol. Biot..

[10] Deahl K., Cooke L., Black L., Wang T., Perez F., Moravec B., Quinn M., Jones R. (2002). Population changes in *Phytophthora infestans* in Taiwan associated with the appearance of resistance to metalaxyl. Pest Manag. Sci..

[11] Hashemi M., Tabet D., Sandroni M., Benavent-Celma C., Seematti J., Andersen C.B., Grenville-Briggs L.J. (2022). The hunt for sustainable biocontrol of oomycete plant pathogens, a case study of *Phytophthora infestans*. Fungal Biol. Rev..

[12] Calvente V., Benuzzi D., de Tosetti M.I.S. (1999). Antagonistic action of siderophores from *Rhodotorula glutinis* upon the postharvest pathogen *Penicillium expansum*. Int. Biodeter. Biodegr..

[13] Zhang Y., Yu X., Zhang W., Lang D., Zhang X., Cui G., Zhang X. (2019). Interactions between endophytes and plants: Beneficial effect of endophytes to ameliorate biotic and abiotic stresses in plants. J. Plant Biol..

[14] Li X.J., Tang H.Y., Duan J.L., Gao J.M., Xue Q.H. (2018). Bioactive alkaloids produced by *Pseudomonas brassicacearum* subsp. *Neoaurantiaca*, an endophytic bacterium from *Salvia miltiorrhiza*. Nat. Prod. Res..

[15] Lastochkina O., Seifikalhor M., Aliniaeifard S., Baymiev A., Pusenkova L., Garipova S., Kulabuhova D., Maksimov I. (2019). Bacillus Spp.: Efficient biotic strategy to control postharvest diseases of fruits and vegetables. Plants.

[16] Brooks S., Klomchit A., Chimthai S., Jaidee W., Bastian A.C. (2022). *Xylaria feejeensis*, SRNE2BP a fungal endophyte with biocontrol properties to control early blight and fusarium wilt disease in tomato and plant growth promotion activity. Curr. Microbiol..

[17] Thomloudi E.E., Tsalgatidou P.C., Baira E., Papadimitriou K., Venieraki A., Katinakis P. (2021). Genomic and metabolomic insights into secondary metabolites of the novel *Bacillus halotolerans* Hil4, an endophyte with promising antagonistic activity against gray mold and plant growth promoting potential. Microorganisms.

[18] Huang X., Ren J., Li P., Feng S., Dong P., Ren M. (2021). Potential of microbial endophytes to enhance the resistance to postharvest diseases of fruit and vegetables. J. Sci. Food Agric..

[19] Asad S., He P., He P., Li Y., Wu Y., Ahmed A., Wang Y., Munir S., He Y. (2021). Interactions between indigenous endophyte *Bacillus subtilis* L1-21 and nutrients inside citrus in reducing Huanglongbing pathogen *Candidatus* Liberibacter Asiaticus. Pathogens.

[20] Munir S., Li Y., He P., He P., He P., Cui W., Wu Y., Li X., Li Q., Zhang S. (2021). Defeating Huanglongbing pathogen *Candidatus* Liberibacter asiaticus with indigenous citrus endophyte *Bacillus subtilis* L1-21. Front. Plant Sci..

[21] Nanjani S., Soni R., Paul D., Keharia H. (2022). Genome analysis uncovers the prolific antagonistic and plant growth-promoting potential of endophyte *Bacillus velezensis* K1. Gene.

[22] Li X.J., Zhang Q., Zhang A.L., Gao J.M. (2012). Metabolites from *Aspergillus fumigatus*, an endophytic fungus associated with *Melia azedarach*, and their antifungal, antifeedant, and toxic activities. J. Agric. Food Chem..

[23] Bilal L., Asaf S., Hamayun M., Gul H., Iqbal A., Ullah I., Lee I.-J., Hussain A. (2018). Plant growth promoting endophytic fungi *Asprgillus fumigatus* TS1 and *Fusarium proliferatum* BRL1 produce gibberellins and regulates plant endogenous hormones. Symbiosis.

[24] Anyasi R.O., Atagana H.I. (2019). Endophyte: Understanding the microbes and its applications. Pak. J. Biol. Sci..

[25] Anyasi R.O., Atagana H.I., Sutherland R. (2019). Comparative study of the colonization of *Chromolaena* and tobacco plants by *Bacteria safensis* CS4 using different methods of inoculation. Pak. J. Biol. Sci..

[26] Zhang J., Islam M.S., Wang J., Zhao Y., Dong W. (2022). Isolation of potato endophytes and screening of *Chaetomium globosum* antimicrobial genes. Int. J. Mol. Sci..

[27] Pinski A., Betekhtin A., Hupert-Kocurek K., Mur L.A.J., Hasterok R. (2019). Defining the genetic basis of plant-endophytic bacteria interactions. Int. J. Mol. Sci..

[28] Rosenblueth M., Martínez-Romero E. (2006). Bacterial endophytes and their interactions with hosts. Mol. Plant-Microbe Interact..

[29] El-Hasan A., Ngatia G., Link T.I., Voegele R.T. (2022). Isolation, identification, and biocontrol potential of root fungal endophytes associated with *Solanaceous* plants against potato late blight *(Phytophthora infestans)*. Plants.

[30] Singh R., Pandey K.D., Singh M., Singh S.K., Hashem A., Al-Arjani A.F., Abd Allah E.F., Singh P.K., Kumar A. (2022). Isolation and characterization of endophytes bacterial strains of *Momordica charantia* L. and their possible approach in stress management. Microorganisms.

[31] La Fua J., Sabaruddin L., Santiaji Bande O., Leomo S., Kade Sutariati G.A., Khaeruni A., Safuan O., Hs G., Corona Rakian T., Muhidin (2021). Isolation of drought-tolerant endophyte bacteria from local tomato plants. Pak. J. Biol. Sci..

[32] Gul Jan F., Hamayun M., Hussain A., Jan G., Iqbal A., Khan A., Lee I.J. (2019). An endophytic isolate of the fungus *Yarrowia lipolytica* produces metabolites that ameliorate the negative impact of salt stress on the physiology of maize. BMC Microbiol..

[33] Wang J.L., Li T., Liu G.Y., Smith J.M., Zhao Z.W. (2016). Unraveling the role of dark septate endophyte (DSE) colonizing maize (Zea mays) under cadmium stress: Physiological, cytological and genic aspects. Sci. Rep..

[34] Ortiz-Urquiza A., Keyhani N.O. (2015). Stress response signaling and virulence: Insights from entomopathogenic fungi. Curr. Genet..

[35] Mizubuti E.S., Aylor D.E., Fry W.E. (2000). Survival of *Phytophthora infestans* sporangia exposed to solar radiation. Phytopathology.

[36] Huang X., You Z., Luo Y., Yang C., Ren J., Liu Y., Wei G., Dong P., Ren M. (2021). Antifungal activity of chitosan against *Phytophthora infestans*, the pathogen of potato late blight. Int. J. Biol. Macromol..

[37] Ren J., Tong J., Li P., Huang X., Dong P., Ren M. (2021). Chitosan is an effective inhibitor against potato dry rot caused by *Fusarium oxysporum*. Physiol. Mol. Plant Pathol..

[38] Liu Y., Liu S., Luo X., Wu X., Ren J., Huang X., Feng S., Lin X., Ren M., Dong P. (2022). Antifungal activity and mechanism of thymol against *Fusarium oxysporum*, a pathogen of potato dry rot, and its potential application. Postharvest Biol. Technol..

[39] Kim M.J., Shim C.K., Park J.H. (2021). Control efficacy of *Bacillus velezensis* AFB2-2 against potato late blight caused by *Phytophthora infestans* in organic potato cultivation. Plant Pathol. J..

[40] Yan H., Qiu Y., Yang S., Wang Y., Wang K., Jiang L., Wang H. (2021). Antagonistic activity of *Bacillus velezensis* SDTB038 against *Phytophthora infestans* in Potato. Plant Dis..

[41] Fan B., Wang C., Song X., Ding X., Wu L., Wu H., Gao X., Borriss R. (2018). *Bacillus velezensis* FZB42 in 2018: The gram-positive model strain for plant growth promotion and biocontrol. Front. Microbiol..

[42] Khalid F., Khalid A., Fu Y., Hu Q., Zheng Y., Khan S., Wang Z. (2021). Potential of *Bacillus velezensis* as a probiotic in animal feed: A review. J. Microbiol..

[43] Cheffi Azabou M., Gharbi Y., Medhioub I., Ennouri K., Barham H., Tounsi S., Triki M.A. (2020). The endophytic strain *Bacillus velezensis* OEE1: An efficient biocontrol agent against Verticillium wilt of olive and a potential plant growth promoting bacteria. Biol. Control.

[44] Hamaoka K., Aoki Y., Suzuki S. (2021). Isolation and characterization of endophyte *Bacillus velezensis* KOF112 from grapevine shoot xylem as biological control agent for fungal diseases. Plants.

[45] Han X., Shen D., Xiong Q., Bao B., Zhang W., Dai T., Zhao Y., Borriss R., Fan B. (2021). The Plant-Beneficial Rhizobacterium Bacillus velezensis FZB42 Controls the Soybean Pathogen Phytophthora sojae Due to Bacilysin Production. Appl. Environ. Microbiol..

[46] Nigris S., Baldan E., Tondello A., Zanella F., Vitulo N., Favaro G., Guidolin V., Bordin N., Telatin A., Barizza E. (2018). Biocontrol traits of *Bacillus licheniformis* GL174, a culturable endophyte of *Vitis vinifera* cv. Glera. BMC Microbiol..

[47] Alfiky A., L’Haridon F., Abou-Mansour E., Weisskopf L. (2022). Disease inhibiting effect of strain *Bacillus subtilis* EG21 and its metabolites against potato pathogens *Phytophthora infestans* and *Rhizoctonia solani*. Phytopathology.

[48] Zhu Y.L., Zhang M.Q., Wang L.S., Mei Y.Z., Dai C.C. (2022). Overexpression of chitinase in the endophyte *Phomopsis liquidambaris* enhances wheat resistance to *Fusarium graminearum*. Fungal Genet. Biol..

[49] Martins J., Verissimo P., Canhoto J. (2022). Isolation and identification of *Arbutus unedo* L. fungi endophytes and biological control of *Phytophthora cinnamomi* in vitro. Protoplasma.

[50] Szekeres A., Kredics L., Antal Z., Kevei F., Manczinger L. (2004). Isolation and characterization of protease overproducing mutants of *Trichoderma harzianum*. FEMS Microbiol. Lett..

[51] Flores A., Chet I., Herrera-Estrella A. (1997). Improved biocontrol activity of *Trichoderma harzianum* by over-expression of the proteinase-encoding gene *prb1*. Curr. Genet..

[52] Chinchilla D., Bruisson S., Meyer S., Zuhlke D., Hirschfeld C., Joller C., L’Haridon F., Mene-Saffrane L., Riedel K., Weisskopf L. (2019). A sulfur-containing volatile emitted by potato-associated bacteria confers protection against late blight through direct anti-oomycete activity. Sci. Rep..

[53] Elsherbiny E.A., Amin B.H., Aleem B., Kingsley K.L., Bennett J.W. (2020). *Trichoderma* volatile organic compounds as a biofumigation tool against late blight pathogen *Phytophthora infestans* in postharvest potato tubers. J. Agric. Food Chem..

[54] Rabbee M.F., Ali M.S., Choi J., Hwang B.S., Jeong S.C., Baek K.H. (2019). *Bacillus velezensis*: A valuable member of bioactive molecules within plant microbiomes. Molecules.

[55] Ongena M., Jacques P. (2008). *Bacillus* lipopeptides: Versatile weapons for plant disease biocontrol. Trends Microbiol..

[56] Stoll A., Salvatierra-Martinez R., Gonzalez M., Araya M. (2021). The role of surfactin production by *Bacillus velezensis* on colonization, biofilm formation on tomato root and leaf surfaces and subsequent protection (ISR) against *Botrytis cinerea*. Microorganisms.

[57] Feng S., Jin L., Tang S., Jian Y., Li Z. (2022). Combination of rhizosphere bacteria isolated from resistant potato plants for biocontrol of potato late blight. Pest Manag. Sci..

[58] Lastochkina O., Baymiev A., Shayahmetova A., Garshina D., Koryakov I., Shpirnaya I., Pusenkova L., Mardanshin I., Kasnak C., Palamutoglu R. (2020). Effects of endophytic *Bacillus Subtilis* and salicylic acid on postharvest diseases (*Phytophthora infestans*, *Fusarium oxysporum*) development in stored potato tubers. Plants.

[59] Buchanan R.E., Gibbons N.E. (1984). Berger Bacterial Identification Manual.

[60] Dong X., Cai M. (2001). Common Bacterial System Identification Manual.

[61] Zhai Y., Zhu J.X., Tan T.M., Xu J.P., Shen A.R., Yang X.B., Li J.L., Zeng L.B., Wei L. (2021). Isolation and characterization of antagonistic *Paenibacillus polymyxa* HX-140 and its biocontrol potential against Fusarium wilt of cucumber seedlings. BMC Microbiol..

[62] Salem E., Naceur D., Olfa T., Adel H., Bacem M., Ridha M., Mohamed S., Ferid L. (2012). Evaluation of antifungal activity from *Bacillus strains* against *Rhizoctonia solani*. Afr. J. Biotechnol..

