# Supplementary material for: Isolation and Screening of Antagonistic Endophytes against Phytophthora infestans and Preliminary Exploration on Anti-oomycete Mechanism of Bacillus velezensis 6-5
Source: Plants (Basel). 2023 Feb 17;12(4):909. doi: 10.3390/plants12040909 (PMC9962363; doi:10.3390/plants12040909)
Supplement: Supplementary file 1 [file plants-12-00909-s001.zip › plants-2209446-supplementary.pdf]

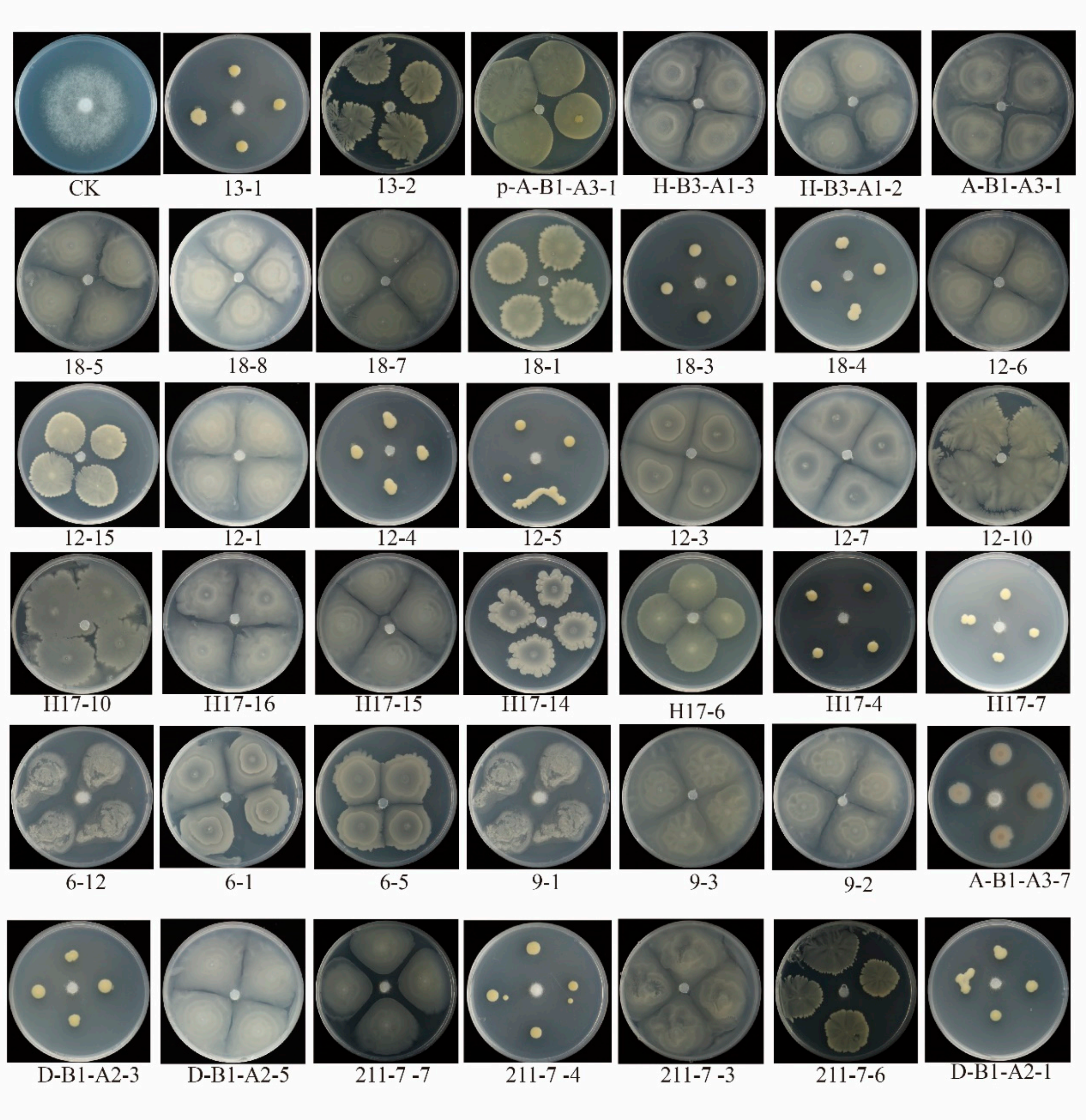


**Figure S1.** Endophytic isolates with antagonistic activity against *Phytophthora infestans* T30-4 *in vitro*.

A

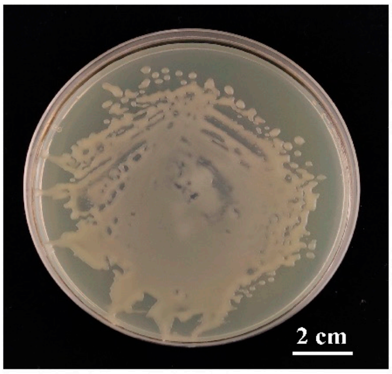


B

| Physiological and biochemical reaction | Result |
|---|---|
| Gram reaction | + |
| Oxygen requirement test | + |
| ethyl red test | - |
| Gelatin liquefaction test | + |
| Catalase test | + |
| $H_2S$ production test | - |
| Starch hydrolysis test | + |
| Nitrate reduction test | + |

C

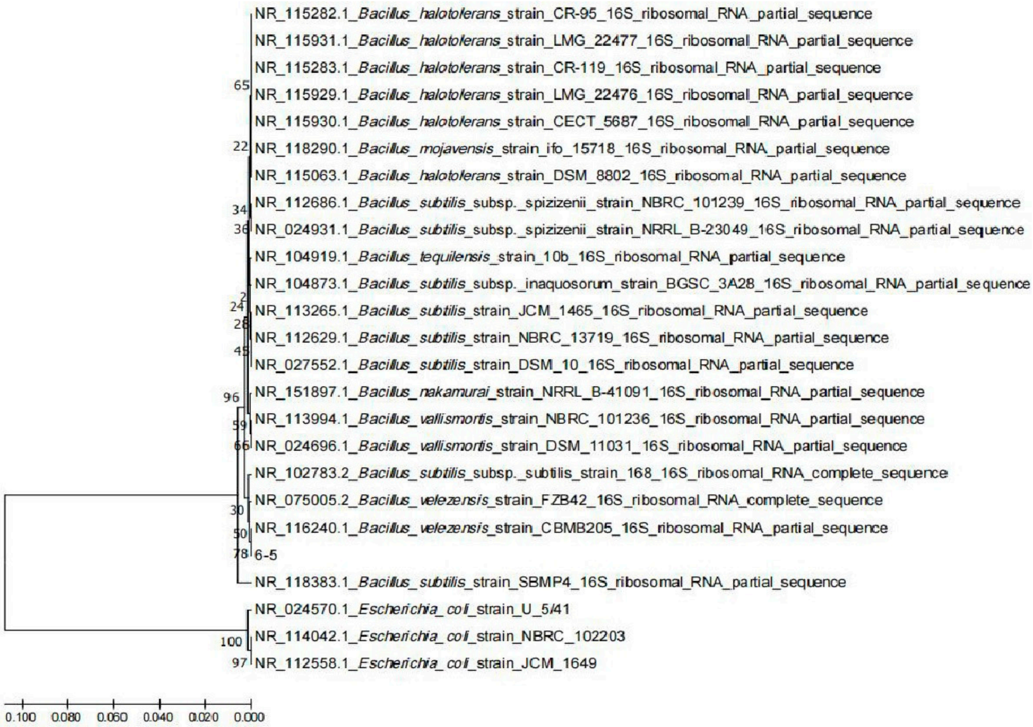


**Figure S2.** Identification of endophyte 6-5. (**A**) Colony characteristics of 6-5; (**B**) Physiological and biochemical characteristics of 6-5, + indicates a positive reaction, - indicates a negative reaction; (**C**) The phylogenetic tree of 6-5.
